# Symptoms and signs in individuals with serology positive for celiac disease but normal mucosa

**DOI:** 10.1186/1471-230X-9-57

**Published:** 2009-07-22

**Authors:** Jonas F Ludvigsson, Lena Brandt, Scott M Montgomery

**Affiliations:** 1 Department of Pediatrics, Örebro University Hospital, Sweden; 2 Clinical Epidemiology Unit, Karolinska University Hospital, Karolinska Institutet, Sweden; 3 Clinical Research Centre, Örebro University Hospital, Sweden; 4 Department of Primary Care and Social Medicine, Charing Cross Hospital, Imperial College, London, UK

## Abstract

**Background:**

Antibody serology is an important tool in the investigation of celiac disease (CD), but does not always correlate with mucosal appearance in the small intestine. Patients with positive CD serology but normal mucosa (Marsh 0) are at increased risk of future CD. In this study we describe a model for identifying and characterizing individuals with normal mucosa but positive CD serology. Such individuals are sometimes referred to as having latent CD.

**Methods:**

The records of ten Swedish pathology departments were used to identify individuals with biopsies indicating normal duodenal/jejunal mucosa. Using the national personal identification number, these data were linked with CD serology data (antigliadin, antiendomysial and tissue transglutaminase antibodies); and we thereby identified 3,736 individuals with normal mucosa but positive CD serology. Two independent reviewers then manually reviewed their biopsy reports to estimate comorbidity. We also randomly selected 112 individuals for validation through patient chart review.

**Results:**

The majority of the 3,736 individuals were females (62%). Children (0–15 years) made up 21.4%. The median number of biopsy specimen was 3. Our review of biopsy reports found that other gastrointestinal comorbidity was rare (inflammatory bowel disease: 0.4%; helicobacter pylori infection: 0.2%). Some 22% individuals selected for patient chart review had a relative with CD. The most common symptoms among these individuals were diarrhea (46%) and abdominal pain (45%), while 26% had anemia. Although 27% of the individuals selected for validation had been informed about gluten-free diet, only 13% were adhering to a gluten-free diet at the end of follow-up.

**Conclusion:**

Individuals with positive CD serology but normal mucosa often have CD-like symptoms and a family history of CD.

## Background

Celiac disease (CD) is an immune-mediated disorder usually confirmed through small-intestinal biopsy[1,2]. In the last 10–15 years, the use of CD serology (antigliadin antibodies (AGA)[3], antiendomysial antibodies (EMA)[4,5], and tissue transglutaminase antibodies (TTGA) [6-8])[9] has changed the diagnostic algorithm for CD [10]. At first, only AGA was used, but recently, this antibody has been largely replaced by EMA and TTGA except in young children. We have earlier shown that 68/68 (100%) Swedish pediatricians and 141/180 (78%) adult gastroenterologist use CD serology in their work-up for CD in at least 90% of individuals with suspected CD[11]. More than 95% of Swedish pediatricians and adult gastroenterologists perform a small intestinal biopsy prior to a diagnosis of CD [11].

In individuals with CD, the biopsy typically shows villous atrophy (VA), crypt hyperplasia and inflammation [12,13]. However, some individuals with positive CD serology have a normal mucosa (Marsh 0). Many of those later develop CD [14-17], and the term "latent CD" is sometimes used when referring to such individuals [18].

We linked Swedish biopsy registers with biochemistry registers to identify individuals with positive CD serology but normal small intestinal mucosa. The purposes of this paper were to (I) describe a model for identifying such patients, (II) describe the symptoms and signs in a subset of patients, and to (III) evaluate if registry-matching is an effective means to identify individuals with positive CD serology but normal small intestinal mucosa.

## Methods

Patients were identified through matching of normal biopsy data (obtained from pathology departments) and data on positive CD serology (obtained from biochemistry departments).

### Classification of biopsy data

To exclude a diagnosis of CD, Swedish pathologists will examine the crypt-villous ratio, the different layers of the intestine, and the number of intraepithelial lymphocytes in small intestinal biopsies. All Swedish pathology departments also use CD3 immunostaining to detect intraepithelial lymphocytosis [11] (classified as inflammation (Marsh 1–2) in Sweden). For a description of the Swedish SnoMed system, and a complete listing of histopathology codes used in this study please see Additional File 1. In the current study, we defined normal mucosa (Marsh 0) [19] as either of the SnoMed codes, M0010 and M0011.

### Collection of biopsy data

**Figure 1:**
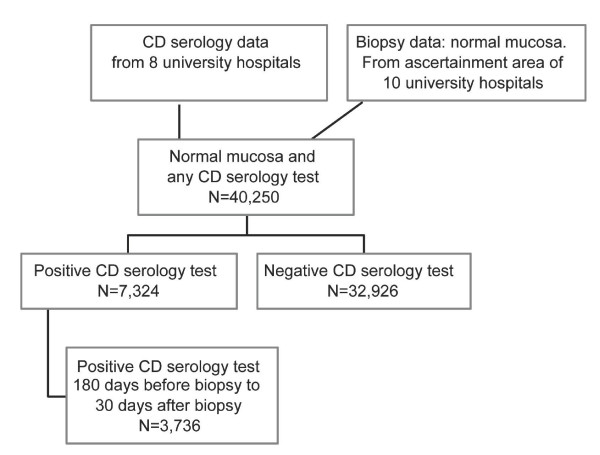
**Identification of individuals with latent CD**. By linkage of biopsy registers and CD serology registers we identified 40,250 individuals with normal mucosa (and no biopsy record of CD or inflammation) with at least one test for CD serology. 7,324 had at some stage a positive CD serology. Some 50% of these (N = 3,736) had a positive CD serology at time of biopsy. 3,285 (87.9% of individuals with latent CD) had positive IgA, while 451 had positive IgG CD serology. Of 3,736 individuals with latent CD, 228 had positive IgA EMA; 3,117 had positive IgA AGA; and 161 had positive IgA TTGA. IgG EMA 1; IgG AGA 491; IgG TTGA 4. Due to overlap this exceeds 3,736). 303 individuals with IgA AGA had at least 2 positive IgA AGA values within the stipulated time frame (≥2 positive tests: IgG AGA 164; IgA TTGA 10).

Biopsy data collection took place between the 27^th ^of October 2006 and the 12^th ^of February 2008. Through computerized searches of all regional pathology departments we obtained 351,403 unique small intestinal biopsies (normal mucosa, inflammation or VA) [11], from 287,586 separate individuals. A detailed description of the data collection has been published earlier [11]. In total there were 244,992 biopsies with normal mucosa. We then identified all individuals who had had a normal biopsy, but never a biopsy with inflammation or VA from ten pathology departments throughout the study period (n = 121,952 individuals)(Figure 1). Using the unique personal identity number (assigned to more than 99% of all Swedish residents) normal biopsy data were matched with CD serology data on a regional basis. The following university hospitals contributed serology data: Karolinska Hospital Solna, Karolinska Hospital Huddinge, Sahlgrenska Hospital, Östra Hospital, Malmö Hospital, Lund Hospital, Uppsala Hospital, and Örebro Hospital. For each CD serology sample we obtained data on date of test, type of test (AGA, EMA, TTGA), antibody levels, IgG/IgA and age-specific reference values at the time of testing. In this study we identified individuals with normal mucosa but positive CD serology up to 180 days before biopsy, and until 30 days after biopsy. Patient data were only included for those who had never had a biopsy showing inflammation or VA (until year 2008). A total of 3,736 individuals fulfilled these criteria (Figure 1).

### Misclassification of individuals identified through registry matching

From among the 3,736 individuals, we randomly selected 120 identified through registry matching (EMA+: n = 40; AGA+: n = 40; TTGA+: n = 40), and requested their patient charts from the department or health care centre that requested the CD serology test, as well as from the department performing the small intestinal biopsy (usually departments of internal medicine/gastroenterology, surgery or pediatrics).

Using individual medical records (and when missing through phone contact with the responsible department/physician), we assessed the proportion of individuals who were correctly identified as having a macroscopically normal mucosa but positive CD serology, and who had never had a biopsy with inflammation or VA).

Again using medical records, we then examined symptoms, signs, laboratory measures, and the extent to which individuals had received information about gluten-free diet. This information was divided according to type of CD serology, and is presented both summarized and weighted according to the distribution of EMA+, TTGA+ and AGA+ among the 3,736 individuals from the complete data-set.

### Comorbidity in biopsy samples

In order to determine if the positive CD serology could be due to other concomitant gastrointestinal disorder (other than pre-stage CD) or systemic disorders with gastrointestinal involvement, we performed a computerized search of the text of the 3,736 biopsy reports with normal mucosa but positive CD serology (see also our earlier paper on VA and inflammation [11]). Thereby we identified 67 biopsy reports where there were indications of comorbidity other than CD. Each of these biopsy reports was then manually screened by two independent reviewers (JFL and research assistant) to confirm or reject the presence of comorbidity. Discrepancies were resolved through a third review of the biopsy reports where necessary.

### Quality assurance and accreditation of CD serology

To explore the quality of CD serology in Sweden we obtained data from the organization Equalis, which has provided laboratory quality assurance in Sweden since 1999 http://www.equalis.se/. The quality assurance scheme can be described briefly as follows. Each year, one of the participating laboratories submits eight blinded CD serology samples to Equalis and a short accompanying clinical description of the individual. From among these eight samples (some positive, some negative for CD serology), Equalis distributes two samples to all participating laboratories for analysis and test data are then reported to Equalis. All assurance data are then merged and presented in aggregated form.

Equalis also provides accreditation of CD serology, and we estimated the proportion of CD serology samples that were accredited and originated from individuals with a small intestinal biopsy (with normal mucosa, inflammation, or VA) in any of the ten participating pathology departments[11]. We then estimated the proportion of accredited CD serology samples from any individual undergoing biopsy in the ascertainment area of these biochemistry departments (independent of the histopathology of the patient).

### Statistics

We calculated 95% confidence intervals (CI). In post-hoc analyses we compared the prevalence of symptoms and signs according to the type of CD serology (EMA vs. AGA vs. TTGA) using the Chi-2 test (Kruskal-Wallis test was used when we examined year of biopsy and age at first biopsy). To decrease the risk of false-positive statistical significance due to multiple testing we used the Bonferroni correction when comparing the patient characteristics according to CD serology subtype [20]; and statistical significance level was set to <0.003 (0.05/18 comparisons) [20].

### Ethics

The current study was performed as part of a larger project on complications in CD. That study was approved by the Regional Ethical Review Board in Stockholm on the 4^th ^of June 2006 (2006/633-31/4) with additional amendments (2007/747-32 and 2008/257-32).

## Results

We identified 3,736 individuals with normal mucosa but positive CD serology (Figure 1). The majority were female and had reached adulthood at first biopsy (Table 1). Most individuals had positive IgA AGA, mirroring the early introduction of AGA in Swedish clinical practice (Table 2). Exact numbers of different antibodies are given in the legend of Figure 1. Some 22% of individuals (95% CI = 15–32%) had a relative with a diagnosis of CD (Table 2). Due to missing data we evaluated misclassification (see below) in 114 individuals; and symptoms and signs in 112 individuals with normal mucosa but positive CD serology.

**Table 1:** Characteristics of individuals with latent celiac disease

	Latent CD
Number	3,736

Age, yrs (median, range)	36; 0–91

Children = 15 years (%)	21.4

Children = 21 years (%)	27.9

Females (%)	62.1

Entry year (median, range)	2001; 1990–2007

**Table 2:** Clinical characteristics of latent CD – patient chart review.

	EMA; N = 38	AGA; N = 36	TTGA; N = 38	Total; N = 112	Total Weighted N = 3,736
**Background data**					

Females	26 (68)	24 (67)	25 (66)	75 (67)	-

Age at first biopsy: median, range (years)	13 (2–80)	34 (1–91)	34 (1–75)	29 (1–91)	-

Year of biopsy, median*	2003	2001	2006	2004	-

Reported heredity for CD	12 (32)	7 (19)	6 (16)	25 (22)	20%

Other diseases					

Diabetes Mellitus, type 1	3 (8)	1 (3)	4 (10)	8 (7)	3%

Depression	1 (3)	4 (11)	8 (21)	13 (12)	11%

Thyroid disease	2 (5)	3 (8)	7 (18)	12 (11)	9%

Liver disease or increased liver enzymes	3 (8)	4 (11)	4 (10)	11 (10)	11%

Symptoms					

Any gastrointestinal symptom#	31 (82)	32 (89)	32 (84)	95 (85)	88%

Diarrhea	16 (42)	18 (50)	17 (45)	51 (46)	49%

Weight loss/growth failure	9 (24)	7 (20)	7 (18)	23 (20)	20%

Abdominal pain	14 (37)	18 (50)	18 (47)	50 (45)	49%

Constipation	10 (26)	4 (11)	6 (16)	20 (18)	12%

Fatigue	6 (16)	6 (17)	5 (13)	17 (15)	16%

Laboratory data					

Anemia and/or iron-deficiency	5 (13)	12 (33)	12 (32)	29 (26)	32%

Folic acid deficiency	2 (5)	0 (0)	4 (10)	6 (5)	1%

B12-deficiency	4 (10)	2 (6)	5 (13)	11 (10)	6%

Erythrocyte sedimentation rate, increased	0 (0)	2 (6)	3 (8)	5 (4)	5%

Percentages are given within brackets. Although latent CD was evaluated in 120 individuals, data on symptoms and signs (± laboratory measures) were only available in 112 individuals (93.3%).

EMA = Endomysial antibodies. AGA = Antigliadin antibodies. TTGA = Tissue transglutaminase antibodies.

Weighted percentages were estimated to mirror the full sample of study participants.

*Only for year of biopsy was there a statistically significant difference according to CD serology subtype when we corrected for multiple testing (Bonferroni correction [20]. There was no statistically significant difference in prevalence of any symptom or sign according to CD serology subtype.

#Weight loss was included among "any GI symptom". Also reflux symptoms (not listed above); nausea and abdominal gases were included among "any GI symptoms".

Additional information: Two individuals had a diagnosis of inflammatory bowel disease (IBD), both were positive for TTGA. Another two individuals had a diagnosis of dermatitis herpetiformis, both were positive for EMA.

### Misclassification of individuals identified through registry matching (n = 114)

**Figure 2:**
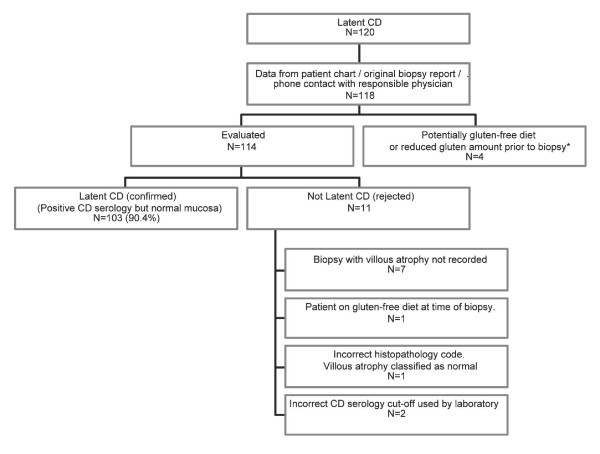
**Overview of individuals with latent CD**. *Three individuals were suspected to have reduced the amount of dietary gluten prior to biopsy (due to other family member with CD). A fourth individual had abstained from gluten at an earlier stage but consumed a normal diet at time of biopsy. The most common reason for misclassification was presence of an earlier unrecorded biopsy with villous atrophy (6.1%).

Through patient chart reviews we were able to confirm that 103/114 (90.4%; 95% CI = 83–95%) individuals identified through our registry matching had normal mucosa but positive CD serology (and no biopsy with inflammation or VA) (Figure 2).

The mean number of biopsy specimens at the first negative biopsy was 2.8 (median = 3). Since several individuals were biopsied on more than one occasion, the estimated mean number of biopsy specimens with normal mucosa collected throughout the entire study period was 3.2.

### Symptoms and signs in individuals with normal mucosa but positive CD serology (n = 112)

Diarrhea was seen in 46% of individuals (95% CI = 36–55%), and constipation in 18% (95% CI = 11–26%). Anemia occurred in 26% of the individuals (95% CI = 18–35%), while B12 deficiency occurred in one out of ten individuals (Table 2). Depression and liver disease were both seen in about 10% of individuals with normal mucosa but positive CD serology. Type 1 diabetes mellitus occurred in 8% of individuals and thyroid disease in 11%.

When we restricted our data to children (n = 39), diarrhea was seen in 49% (95% CI = 32–65%), weight loss/growth failure in 38% (95% CI = 23–55%), constipation in 28% (95% CI = 15–45%), and anemia in 18% (95% CI = 8–34%). There were no statistically significant differences in symptoms and signs in comparisons using the various measures of CD serology, EMA+, AGA+, TTGA+, (data not shown).

### Information about gluten-free diet

Some 25% of the individuals with normal mucosa but positive CD serology had received information about gluten-free diet from a physician or a dietician (Table 3). More individuals with positive EMA than positive AGA or TTGA had received information about gluten-free diet, although these differences are not statistically significant after correction for multiple testing (data not shown).

**Table 3:** Gluten-free diet in latent CD (%).

	EMA; N = 38	AGA; N = 36	TTGA; N = 38	Total; N = 112	Total Weighted N = 3,736
Informed about gluten-free diet by physician or dietician	17 (45)	5 (14)	8 (21)	30 (27)	16%

Clinical response to gluten-free diet*	9/17 (53)	2/5 (40)	4/8 (50)	15/30 (50)	41%

Gluten-free diet at end of follow-up	10 (26.3)	1 (3)	4 (11)	15 (13)	5%

This table shows the proportion of individuals with latent CD that were encouraged to follow a gluten-free diet at some stage, as well as the proportion of individuals on a gluten-free diet at the end of follow-up (until year 2008).

* Percentages were calculated based on the number of individuals receiving information about gluten-free diet.

However, only 13% (95% CI = 8–21%) were on a gluten-free diet at the end of follow-up of this study (year 2008).

### Comorbidity in biopsy samples

Diagnosed comorbidity was rare. There were 14 diagnoses of IBD (in 3,736 patients; i.e. 0.4%; 95% CI = 0.2–0.4%)(Table 4). Helicobacter pylori infection was mentioned in 9 biopsy reports (0.2%; 95% CI = 0.1–0.5%).

**Table 4:** Comorbidity in 67 biopsy samples – Results of manual examination

Histopathology	Normal/Latent CD
**Samples, No.**	3,736 (%)

	

Gastric metaplasia	4 (0.1)

Helicobacter pylori infection	9 (0.2)

Mb Crohn	1 (<0.1)

Colitis: Microscopic/Ulcerative	11 (0.3)

Any IBD*	14 (0.4)

We also searched plain biopsy text for autoimmune enteropathy, giardiasis, inflammatory granuloma, lymphoma, other cancer, tropical sprue, postoperative changes, systemic lupus Erythematosus, vasculitis, Whipple's disease, immune deficiency, Behcet's disease, graft vs. host disease/transplantation, sarcoidosis, immunoglobuline disease and Zollinger-Ellison Disease. For these disorders, the computerized search only yielded results that were rejected on manual examination.

*Includes biopsy reports where non-specific IBD was listed.

### Accreditation of CD serology

Almost all IgA serology in individuals undergoing small intestinal biopsy had been accredited (Table 5). However, there is no accreditation of IgG CD serology in Sweden. As part of the accreditation procedure, CD serology is tested in a blinded manner in the biochemistry department seeking accreditation. The validation and accreditation is carried out by the non-profit company Equalis http://www.equalis.se/. In 2007, the participating laboratories were sent one positive and one negative sample, and showed high consistency between different laboratories: IgA AGA (17/17 positive samples were detected and 14/14 negative samples were correctly assessed), IgA EMA (18/19 and 4/4) and IgA TTGA (18/19 and 17/18). The laboratories themselves chose the appropriate assay. It is likely that several laboratories performed the transglutaminase IgA first, and finding it negative in "sample 2" did not perform the IgA EMA test (which was tested in only 4 laboratories).

**Table 5:** CD serology – Accreditation of laboratories.

Laboratory	Analyses	Accreditation Date	Number of serology tests	Accredited proportion*
	Endomysial (IgA)	29-jan-98	6,755	95.5
	
**Örebro**	Gliadin (IgA)	29-jan-98	3,737	91.9
	
	Transglutaminase (IgA)	19-dec-01	2,974	95.7

**Karolinska Solna and Huddinge§**	Endomysial (IgA)	1996	8,243	98.0#
	
	Gliadin (IgA)	1995	28,422	95.7#
	
	Transglutaminase (IgA)	2002	20,042	93.4#

**Sahlgrenska and Östra Hospitals§**	Endomysial (IgA)	1997	10,855	98.4#
	
	Gliadin (IgA)	1997	17,426	72.2#
	
	Transglutaminase (IgA)	12-jun-03	1,100	100.0

**Lund and Malmö Hospitals§**	Endomysial (IgA)	19-dec-96	18,884	91.9
	
	Gliadin (IgA)	19-dec-96	14,910	79.5
	
	Transglutaminase (IgA)	2001	2,268	99.8#

**Uppsala**	Gliadin (IgA)	1999	7,166	33.8#
	
	Endomysial (IgA)	1999	3,470	65.1#
	
	Transglutaminase (IgA)	1999	1,675	100#

*Proportion of IgA serology samples analyzed after accreditation date. IgG serology samples are not accredited in Sweden.

§Accreditation dates were the same for laboratories from the same university city/region (listed together above).

#When estimating the proportion of IgA CD serology samples analyzed after accreditation date, we have assumed that accreditation took place on the 1^st ^of July the relevant year.

## Discussion

Through biochemistry registers and biopsy registers we were able to identify 3,736 individuals with normal mucosa but positive CD serology. Such individuals are at increased risk of future CD[15,21]. Our patient chart review found that more than 90% of individuals identified through matching of biopsy registers and biochemistry registers were correctly identified. Previous data suggest that normal mucosa (Marsh 0) is very seldom misclassified (in a blinded test, 96% of samples with normal mucosa were correctly classified by Swedish pathologists [11]). In addition all surveyed Swedish pathology departments use CD3-immunostaining to detect intraepithelial lymphocytes[11]. Most Swedish pathology departments consider >30 intraepithelial lymphocytes per 100 as abnormal[11] (and not >40 as was suggested in older literature[12]). Recent data indicate that a cut-off of 25 per 100[22] or 30 per 100 is appropriate [23].

The symptoms and signs of the 112 individuals whose patient charts were reviewed in this study were similar to those seen in CD [11,24,25]. This is however unsurprising, since most individuals in our cohort had probably been tested for CD serology and undergone small intestinal biopsy due to suspected CD. Earlier studies have found a varying prevalence of diarrhea in individuals with CD (Green: 85%[26]; Ciacci 60%[24]; Ludvigsson (children) 53%[27]; Fasano 35%[28]). This compares with 45% in the current study of individuals with a normal mucosa.

Some 85% of individuals in this study reported at least some kind of gastrointestinal symptoms. This was slightly higher proportion than in the study by Salmi et al (18/25 individuals (72%) with positive CD serology and Marsh 0–1 in their study had "abdominal symptoms")[16], but then we also included weight loss in our definition of gastrointestinal symptoms. The prevalence of constipation in the current study was almost identical to that of individuals with screening-detected CD in the Fasano et al study [28]. In a British study, 50% of individuals with undiagnosed CD had anemia on presentation [29], and this may partly be due to occult gastrointestinal bleeding [30]. Occult bleeding is however related to the degree of mucosal injury[30], and that may explain the lower rate of anemia in our population since they all had Marsh 0. Of our individuals, one in four had anemia and/or iron deficiency. Of note, we found no difference in the prevalence of symptoms and signs according to the type of CD serology (EMA+, AGA+, TTGA+).

Twenty-two percent of validated individuals had a family history of CD. This is consistent with data from an American study of adults with *diagnosed *CD [26], where 19% reported having a relative with CD. There are several studies suggesting that individuals with CD are at increased risk of having a relative with a diagnosis of CD [28,31,32], but to our knowledge ours is the first study to show this in individuals with normal mucosa but positive CD serology.

The specificity of CD serology is high [16], and a negative CD serology will almost always rule out a diagnosis of CD [33]. However the positive predictive specificity (PPV) of CD serology is not infallible, with two recent papers showing a PPV for TTGA of 29% [33] and 76% [20] respectively. This does not mean that positive CD serology in individuals with normal mucosa is of no clinical significance. Many individuals with positive CD serology but normal mucosa will go on to develop VA [14-17]; and IgA AGA+[34], EMA+[34], and TTGA+[35] are all associated with increased mortality.

Due to the historical nature of this study, AGA was the only available screening tool for much of the study period (beginning in 1990). AGA has lower specificity for CD than TTGA and especially EMA[36]. Although we cannot rule out the possibility that some individuals with positive CD serology and normal mucosa do not suffer from a raised risk for future CD it should be emphasized that the specificity of IgA AGA is nevertheless above 80% in adults [36] (around 90% in a later report by Rostom et al [2]). In children it may be even higher [36] (and more than 20% of our population consisted of children aged ≤15 years). These antibody tests were not performed as part of screening for CD in the general population but likely due to symptoms that also merited a small intestinal biopsy. Still, it should be remembered that not all individuals with positive CD serology develop CD [16], and that positive CD serology can sometimes be transient.

We chose to include individuals with positive IgG and normal mucosa in this study. IgA deficiency is strongly associated with CD [37-39], and in these individuals IgG is a frequently used screening tool for CD; and all individuals in our study had undergone small intestinal biopsy. Although IgG EMA and TTGA are associated with CD [40], less is known of the specificity and positive predictive value of IgG AGA.

Another potential limitation of our study is that some biopsy samples may have been misclassified. Small intestinal inflammation is sometimes missed by pathologists [11], and in patchy VA[41] and inflammation, several biopsies are needed to confirm a CD diagnosis [42,43]. Our study was based on historical data, when the use of capsule biopsy was widespread (with fewer biopsy specimen as a consequence), especially among pediatricians [11]. On average, 3 biopsy specimens had been obtained from each individual, and some of the individuals may have had a false-negative patchy VA. Neither can we rule out that some individuals classified as having Marsh 0 had in fact sub-microscopic changes (microscopic enteritis), and we lack individual-based data on immunohistochemistry for our individuals [44,45]. Microscopic enteritis has increasingly been recognized as an important cause of CD-like symptoms including malabsorption [44]. These individuals have a low count of intraepithelial lymphocytes but nevertheless show altered enterocytes and affected microvilli [44]. Microscopic enteritis is also associated with other autoimmune diseases.

Among individuals with normal mucosa but positive CD serology and available data, we also characterized the use of gluten-free diet. Approximately one individual in four had received information about gluten-free diet from a physician or from a dietician after having their biopsy. However, some individuals with positive CD serology may have had tried a gluten-free diet themselves, without prior prescription. Among individuals on a gluten-free diet, about half reported a clinical response. It has previously been shown that symptoms in individuals with minor mucosal abnormalities may improve on a gluten-free diet [46]. This supports our belief that at least some individuals with normal mucosa but positive CD serology have early stage CD or a phenotype of CD.

## Conclusion

Individuals with positive CD serology but normal mucosa often have CD-like symptoms and a family history of CD. Such individuals may be identified through registry matching.

A proportion of individuals with normal mucosa (Marsh 0) and positive CD serology may in fact have microscopic enteritis[44]. Earlier research has shown that individuals with macroscopically normal mucosa but positive CD serology often progress to Marsh I-III with symptoms consistent with CD[15,21].

## Abbreviations

AGA: antigliadin antibodies; CD: celiac disease; EMA: endomysium antibodies; TTGA: Tissue transglutaminase antibodies; VA: villous atrophy.

## Competing interests

The authors declare that they have no competing interests.

## Authors' contributions

JFL designed the study, wrote the ethics application, collected the data, analyzed the statistics, and wrote the manuscript. LB helped JFL manage the data, and critically reviewed the manuscript. SM critically reviewed the manuscript and contributed to the study design. All authors read and approved the final manuscript

## Pre-publication history

The pre-publication history for this paper can be accessed here:

http://www.biomedcentral.com/1471-230X/9/57/prepub

## Supplementary Material

Additional file 1 Table – Small intestinal histopathology classifications – a comparison. Click here for file
