# Supplementary material for: Symptoms and signs in individuals with serology positive for celiac disease but normal mucosa
Source: BMC Gastroenterol. 2009 Jul 22;9:57. doi: 10.1186/1471-230X-9-57 (PMC2724402; doi:10.1186/1471-230X-9-57)
Supplement: Additional file 1 — Table – Small intestinal histopathology classifications – a comparison. [file 1471-230X-9-57-S1.doc]

**Additional file 1**

**Table - Small intestinal histopathology classifications – a comparison**

| ***Classification used in this project*** | ***Normal*** | ***Inflammation*** | | ***Villous atrophy (VA)*** | | |
| --- | --- | --- | --- | --- | --- | --- |
| Marsh Classification* | Type 0 | Type 1 | Type 2 | Type 3a | Type 3b | Type 3c |
| Marsh  Description | Pre-infiltrative | Infiltrative | Infiltrative-hyperplastic | Flat destructive | | |
| Corazza et al | - | Grade A | | Grade B1 | | Grade B2 |
| SnoMed Codes | M0010, M0011 | M40000, M41000, M42000, M43000, M47000, M47170 | | M58,  D6218,  M58005 | M58,  D6218,  M58006 | M58,  D6218,  M58007 |
| KVAST/Alexander classification | I  Normal | II  Intraepithelial lymphocytosis (IEL)# | | III  Partial VA | IV  Subtotal VA | IV  Total VA |
|  |  |  |  |  |  |  |
| *Characteristics* |  |  |  |  |  |  |
| Villous atrophy | - | - | - | + | ++ | ++ |
| IEL# | - | + | + | + | + | + |
| Crypt hyperplasia | - | - | + | + | ++ | ++ |

*We have not included Marsh type 4 in this classification since such lesions are very rare (Dickson et al) and cannot be identified through SnoMed Codes. For a description of partial, subtotal and total villous atrophy, we refer to the papers by Rostami et al and Rostom et al.

# Increased intraepithelial lymphocyte count (often >30/100 epithelial cells) (Veress et al).

Corazza GR, Villanacci V, Zambelli C, Milione M, Luinetti O, Vindigni C, Chioda C, Albarello L, Bartolini D, Donato F: Comparison of the interobserver reproducibility with different histologic criteria used in celiac disease. *Clin Gastroenterol Hepatol* 2007, 5(7):838-843.

Dickson BC, Streutker CJ, Chetty R: Coeliac disease: an update for pathologists. *J Clin Pathol* 2006, 59(10):1008-1016.

Veress B, Franzen L, Bodin L, Borch K: Duodenal intraepithelial lymphocyte-count revisited. *Scand J Gastroenterol* 2004, 39(2):138-144.

Rostom A, Murray JA, Kagnoff MF: American Gastroenterological Association (AGA) Institute technical review on the diagnosis and management of celiac disease. *Gastroenterology* 2006, 131(6):1981-2002.

Rostami K, Villanacci V: Microscopic enteritis: novel prospect in coeliac disease clinical and immuno-histogenesis. Evolution in diagnostic and treatment strategies. *Dig Liver Dis* 2009, 41(4):245-252.
